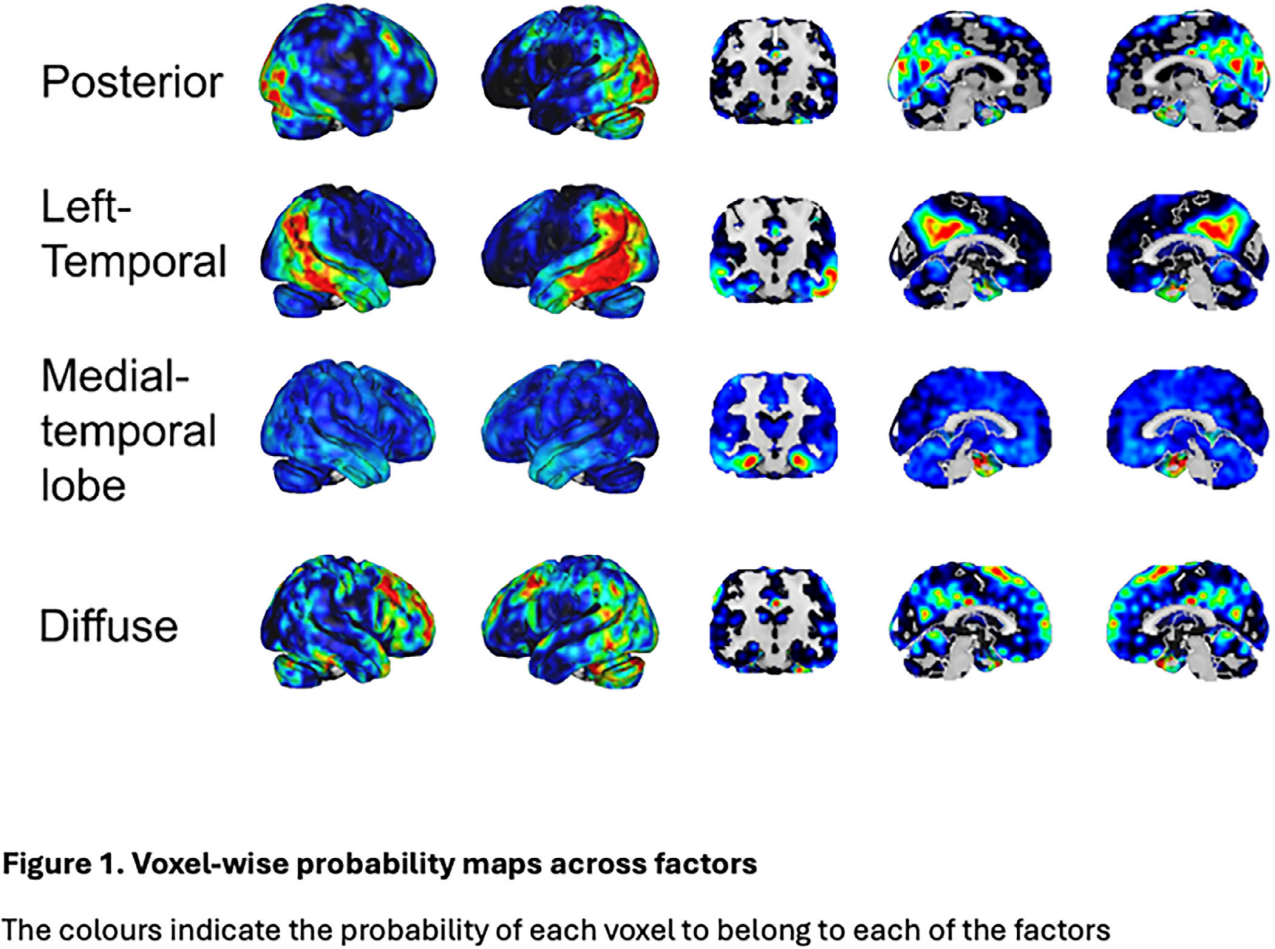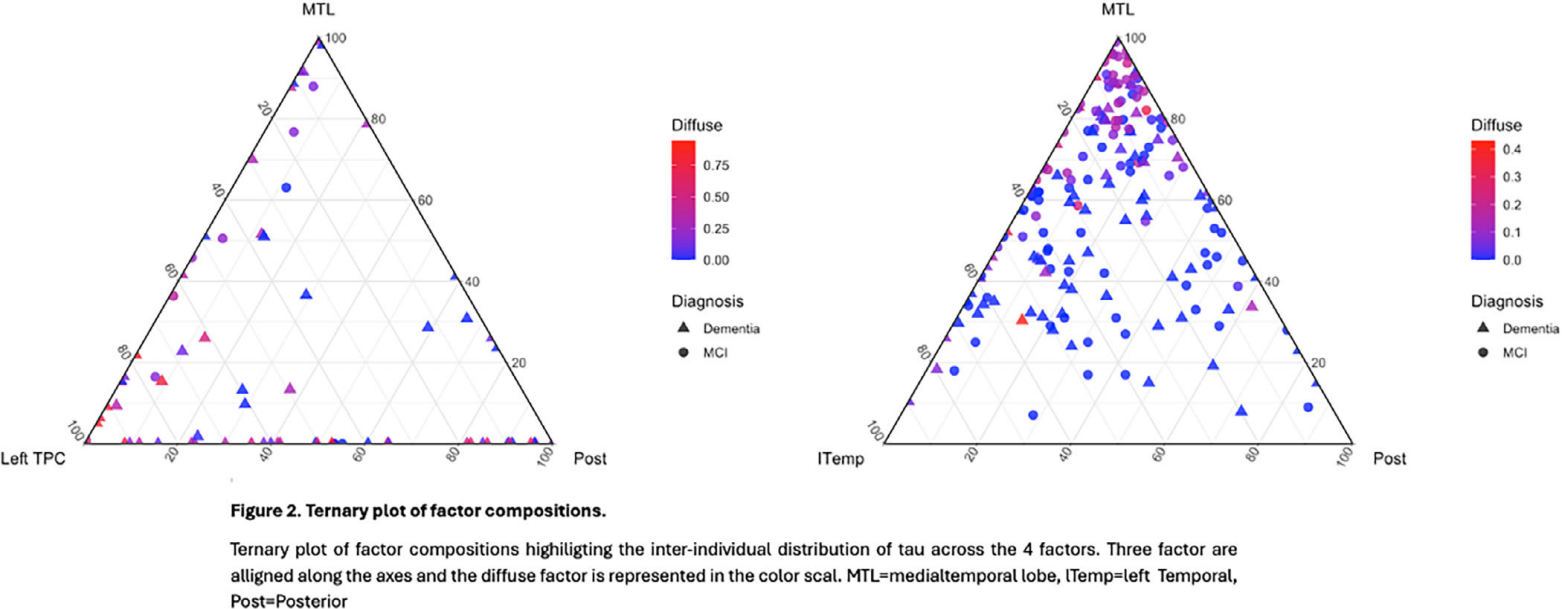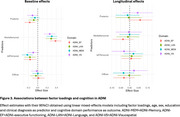# Bayesian modelling applied to Tau PET to explore the effects of heterogeneity in tau patterns

**DOI:** 10.1002/alz70856_105595

**Published:** 2026-01-08

**Authors:** Ye Xia, Keith A. Johnson, Georges El Fakhri El Fakhri, Nicolas J Guehl, Elsmarieke van de Giessen, Wiesje M. van der Flier, Yolande A.L. Pijnenburg, Rik Ossenkoppele, Colin Groot

**Affiliations:** ^1^ Massachusetts General Hospital, Melrose, MA, USA; ^2^ Alzheimer Center Amsterdam, Department of Neurology, Amsterdam UMC, Amsterdam Neuroscience, Netherlands., Amsterdam, Amsterdam, Netherlands; ^3^ Gordon Center for Medical Imaging, Massachusetts General Hospital, Harvard Medical School, Boston, MA, USA; ^4^ Department of Radiology and Biomedical Imaging, Yale School of Medicine, New Haven, CT, USA, New Haven, CT, USA; ^5^ Department of Biomedical Informatics and Data Science, Yale School of Medicine, New Haven, CT, USA, New Haven, CT, USA; ^6^ Yale School of Medicine, New Haven, CT, USA; ^7^ Department of Radiology and Nuclear Medicine, Amsterdam UMC, Vrije Universiteit Amsterdam, Amsterdam Neuroscience, Amsterdam, Netherlands; ^8^ Amsterdam Neuroscience, Brain Imaging, Amsterdam, Netherlands; ^9^ Alzheimer Center Amsterdam, Neurology, Vrije Universiteit Amsterdam, Amsterdam UMC location VUmc, Amsterdam, Netherlands; ^10^ Alzheimer Center Amsterdam, Neurology, Vrije Universiteit Amsterdam, Amsterdam UMC location VUmc, Amsterdam, Amsterdam, Netherlands; ^11^ Alzheimer Center Amsterdam, Department of Neurology, Amsterdam Neuroscience, Vrije Universiteit Amsterdam, Amsterdam UMC, Amsterdam, Netherlands; ^12^ Clinical Memory Research Unit, Lund University, Lund, Lund, Sweden

## Abstract

**Background:**

Traditionally, mutually exclusive subgroups have been used to explore the effects of tau heterogeneity on cognitive outcomes, which ignores much of the complexity and overlap found in tau patterns across individuals.

**Method:**

We used a data‐driven Bayesian modeling framework based on Latent Dirichlet Allocation to identify 4 tau patterns (i.e., factors) in a discovery cohort of amyloid‐positive individuals with symptomatic AD from the Amsterdam Dementia Cohort (*N* = 93, age=65.26, Females=43, education=10y Dementia=81, MCI=12). For an independent replication sample from ADNI (*N* = 191, age=73.4, Females=86, education = 15.87, Dementia=74, MCI=117), we then extracted factor loadings across each of the factors, which indicate to what extent an individual's tau pattern is represented by each of the factors. Inter‐individual distributions of factor loadings are interdependent and add up to 100. In ADNI, we then employed linear‐mixed effects analyses adjusted for age, sex, education and clinical diagnosis, to assess the associations between factor loading and (longitudinal) cognitive performance (ADNI‐Memory [MEM], ADNI‐executive functioning [EF], ADNI‐Language [LAN], and ADNI‐Visuospatial [VS]).

**Result:**

The most optimal solution applied to the ADC cohort yielded four distinct but partly overlapping tau factors, which were named; posterior, left temporal, diffuse, and medial temporal lobe (Figure 1). Figure 2 displays the distribution of factor loadings in ADC and ADNI, highlighting that ADNI participants generally load high on the medial temporal lobe factor, consistent with limbic‐predominant tau pathology often found in late‐onset AD. We found that higher factor loading on the medial temporal factor (rather than the neocortical factors) was related to better baseline cognitive performance across all domains (β range:0.17‐0.39, all *p* <0.05). High loading on the posterior factor was associated with worse baseline executive functioning (β = ‐0.17, *p* =  0.00) and worse visuospatial functioning (β = ‐0.11, *p* =  0.05). Left temporal loading was related to worse executive functioning (β = ‐0.16, *p* =  0.02), memory (β = ‐0.2, *p* =  0.00) and language functioning (β = ‐0.15, *p* =  0.01) (Figure 3)

**Conclusion:**

Our data driven method identified biologically plausible latent tau PET patterns that could be applied to an independent dataset and our results highlight that neocortical factor loading is related to a non‐amnestic clinical profile.